# Reintervention for recurrent biliary obstruction after stent-in-stent deployment of multi-hole self-expandable metal stents

**DOI:** 10.1055/a-2534-3143

**Published:** 2025-02-20

**Authors:** Takeshi Ogura, Yuki Uba, Takafumi Kanadani, Nobuhiro Hattori, Hiroki Nishikawa

**Affiliations:** 1 Endoscopy Center, Osaka Medical and Pharmaceutical University Hospital, Takatsuki, Japan; 2 2nd Department of Internal Medicine, Osaka Medical and Pharmaceutical University, Takatsuki, Japan

Malignant hilar biliary obstruction (MHBO) can be treated by bilateral deployment of uncovered self-expandable metal stents (SEMSs) using a stent-in-stent (SIS) technique 1 . If an uncovered SEMS becomes obstructed due to tumor ingrowth, several steps, such as guidewire insertion, stricture dilation, or stent deployment, are needed. In addition, stent obstruction is sometimes observed, and because of technical complexity, some authors perform endoscopic ultrasound-guided biliary drainage; however, it is well known that critical adverse events such as stent migration into the abdominal cavity can occur with this approach. Recently, a fully covered SEMS with multiple side holes (MHSEMS; HANAROSTENT Biliary Multi-hole NEO; M.I. Tech Co., Ltd, Pyeongtaek, South Korea) has become available ( Fig. 1 ). This stent was designed to prevent stent migration by allowing small tissue ingrowths to form in the multiple small (1.8 mm) side holes along the covering membrane 2 3 . The primary indication for this stent is distal biliary obstruction, but biliary stenting for MHBO using an SIS technique has also been reported 4 . However, a reintervention technique for MHSEMS obstruction after SIS deployment has not been reported. Here, we describe technical tips for reintervention using the SIS technique for an occluded MHSEMS.

**Fig. 1:**
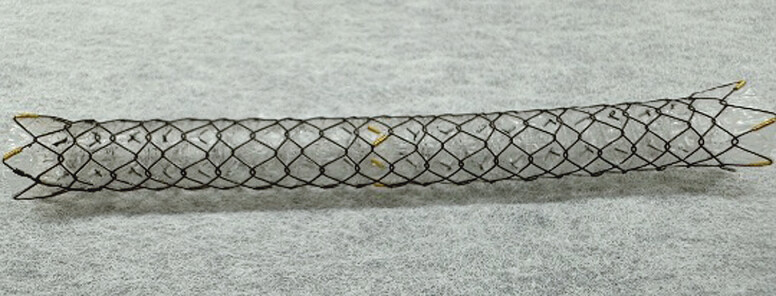
Fully covered self-expandable metal stent with side holes (HANAROSTENT Biliary Multi-hole NEO; M.I. Tech Co., Ltd, Pyeongtaek, South Korea).

An 83-year-old man was admitted to our hospital due to MHBO. He had undergone an SIS technique using MHSEMSs 6 months earlier. However, acute cholangitis developed, and reintervention was attempted. First, a guidewire was deployed into the biliary tract. Cholangiography showed no tumor ingrowth, but right hepatic bile duct stenosis, which might have been due to tumor spread, was observed ( Fig. 2 **a** ). Stent removal was then attempted using biopsy forceps. The proximal end of the MHSEMS was grasped ( Fig. 2 **b** ) and the stents were successfully removed through the scope ( Fig. 2 **c** ). As no tumor ingrowth was observed, reintervention was easily performed ( Fig. 2 **d** ). SIS deployment using MHSEMS was successfully performed without any adverse events ( Video 1 ). After reintervention, no adverse events, including recurrent biliary obstruction, were observed until the patient’s death (4 months).

**Fig. 2:**
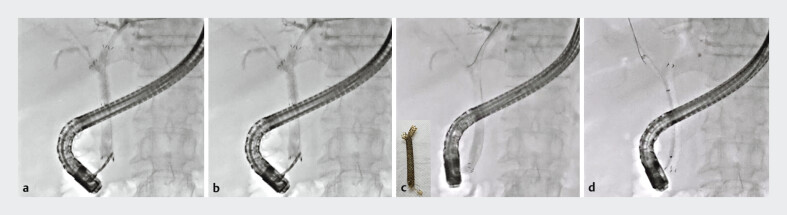
Cholangiographic images showing stent removal and reintervention. **a** Cholangiography showed no tumor ingrowth, but right hepatic bile duct stenosis, which might have been due to tumor spread, was observed. **b** The proximal end of the fully covered self-expandable metal stent with side holes (MHSEMS) was grasped. **c** The stents were successfully removed through the scope. **d** Reintervention was performed by stent-in-stent deployment using MHSEMSs.

Reintervention for recurrent biliary obstruction after stent-in-stent deployment of multi-hole self-expandable metal stents.Video 1

In conclusion, an SIS technique using MHSEMSs may prevent stent ingrowth, and because the stents can be removed, reintervention may be easily performed.

Endoscopy_UCTN_Code_TTT_1AR_2AZ

## Notes

Endoscopy E-Videos https://eref.thieme.de/e-videos
*E-Videos* is an open access online section of the journal *Endoscopy* , reporting on interesting cases and new techniques in gastroenterological endoscopy. All papers include a high-quality video and are published with a Creative Commons CC-BY license. Endoscopy E-Videos qualify for HINARI discounts and waivers and eligibility is automatically checked during the submission process. We grant 100% waivers to articles whose corresponding authors are based in Group A countries and 50% waivers to those who are based in Group B countries as classified by Research4Life (see: https://www.research4life.org/access/eligibility/ ). This section has its own submission website at https://mc.manuscriptcentral.com/e-videos .
